# Ground Speed Optical Estimator for Miniature UAV

**DOI:** 10.3390/s21082754

**Published:** 2021-04-13

**Authors:** Piotr Chmielewski, Krzysztof Sibilski

**Affiliations:** 1 Institute of Aeronautics and Applied Mechanics, Faculty of Power and Aeronautical Engineering, Doctoral School No. 4, Warsaw University of Technology, 00-665 Warsaw, Poland; piotr.chmielewski.dokt@pw.edu.pl; 2 Institute of Aeronautics and Applied Mechanics, Faculty of Power and Aeronautical Engineering, Warsaw University of Technology, 00-665 Warsaw, Poland

**Keywords:** drift estimation, template matching, optical sensor, UAV, MUAV

## Abstract

In a conventional Unmanned aerial vehicles (UAV) navigational system Global Navigation Satellite System (GNSS) sensor is often a main source of data for trajectory generation. Even video tracking based systems need some GNSS data for proper work. The goal of this study is to develop an optics-based system to estimate the ground speed of the UAV in the case of the GNSS failure, jamming, or unavailability. The proposed approach uses a camera mounted on the fuselage belly of the UAV. We can obtain the ground speed of the airplane by using the digital cropping, the stabilization of the real time image, and template matching algorithms. By combining the ground speed vector components with measurements of airspeed and altitude, the wind velocity and drift are computed. The obtained data were used to improve efficiency of the video-tracking based on a navigational system. An algorithm allows this computation to be performed in real time on board of a UAV. The algorithm was tested in Software-in-the-loop and implemented on the UAV hardware. Its effectiveness has been demonstrated through the experimental test results. The presented work could be useful for upgrading the existing MUAV products (with embedded cameras) already delivered to the customers only by updating their software. It is especially significant in the case when any necessary hardware upgrades would be economically unjustified or even impossible to be carried out.

## 1. Introduction

Unmanned aerial vehicles (UAV), or, in this case, more precisely autonomous Miniature Unmanned Aerial Vehicles (MUAV) are increasingly common in our lives. They are used in both civil application and for military purpose. Often they are able to autonomously track a target, without a necessity to be piloted. These features are used for example for taking video in an autonomous mode [1,2]. Unfortunately, video tracking capabilities are not sufficient for navigating and controlling an MUAV. An autopilot can combine data from the inertial measurement unit (IMU), attitude and heading reference system (AHRS), Global Navigation Satellite System (GNSS), LIDAR, or video-tracker to generate a proper trajectory of the flight [3,4]. Less data delivered to the autopilot results in less precise trajectory generation. GNSS is one of the most crucial data sources for autopilot. Without these data, the navigational capabilities of the MUAV are severely diminished. An MUAV that has lost its position data still can video track the target, but its trajectory will not adapt to the drift caused by the wind. This is really important particularly in scenarios where GNSS jamming is common practice or in areas where GPS data are not available at all.

Let us consider a scenario where fixed-wing MUAV is used to autonomously video track a selected target. Let us assume that GNSS data are not available. To perform successful navigation in flight by using a video tracker without knowing the precise position, we will need to replace GNSS information with another source of data. There are many techniques for achieving that. IMU-based Dead Reckoning [5] is first of all. The main issue with that approach is that a digital IMU, which is used in MUAV, has a significant error that is constantly growing during the flight [6,7]. More precise units are either too expensive or too big and heavy [8]. A lot of MUAV are cheap platforms created for a wider audience, like hobbyists. The embedded hardware cost must be adequate for the total cost of the platform itself. Some studies aimed to use Dead Reckoning, combined with imaging techniques [9] or Artificial Intelligence (AI) [10], to minimize an error in position estimation. Both of the mentioned techniques were used to estimate the position, velocity, and attitude of vehicle with some success. Another approach is to use a combination of computer vision techniques with deep learning [11,12,13,14]. According to [11], vision based systems can be roughly classified into three categories: map-based, map-building, and map-less systems. The first category includes global localization, encompassing incremental localization to estimate the position on the stored map. The second one concerns simultaneous localization and mapping (SLAM), which is able to create a map in real time and estimate the position in relation to the known reference point. The third group uses feature tracking and optical flow to only navigate by extracting distinct features in the observed environment [15]. The aerospace industries developed map-less systems that can estimate the ground speed while using embedded cameras [16]. These are using deep learning to track runway characteristic points and by calculating a change of their position on the image they are able to estimate the ground speed of the vehicle. These systems are used to perform fully automatic start and landing procedures, but they are complicated and they have high calculating consumption.

**Figure 1:**
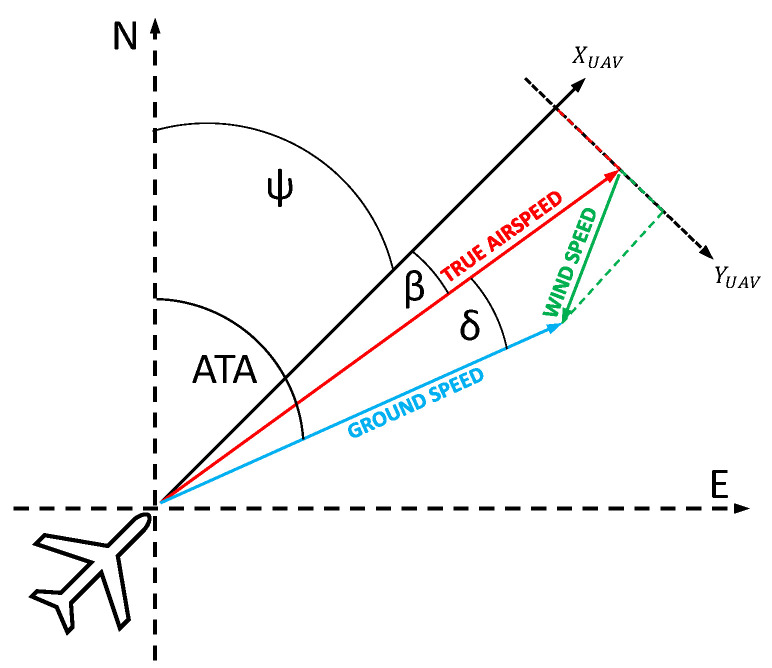
Formation of the expanded wind triangle; the figure includes the sideslip angle β. This angle is nonzero when for example the aircraft is not flying straight ahead relative to the aircraft coordinate system, the reference system is not well aligned or turbulent wind fluctuations are present.

**Figure 2:**
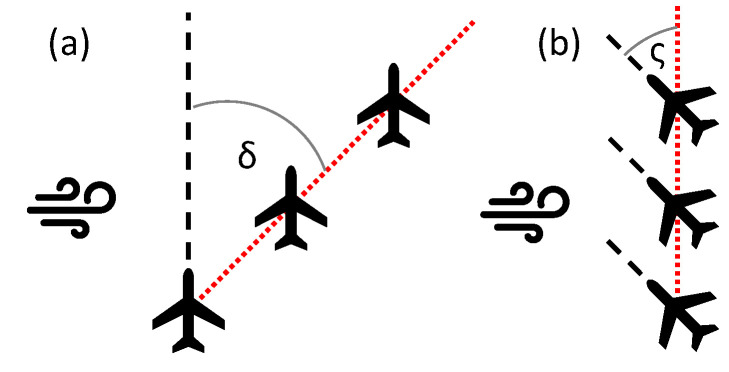
Wind effect to the aircraft. (**a**) Drifting effect of the wind δ-drift angle. (**b**) Compensating the drift by crab flight ς-crab-angle (correction angle).

However, what if we do not really need this amount of data? In video-tracking navigation, where our goal is to hit the observed target, what we need is just the target position on the camera matrix, the vehicle orientation in space, and the lateral displacement of the aircraft caused by the wind. The flight path may differ from the planned one, due to the wind factor. This difference is called the drift, and it is the angle between the true airspeed vector and the ground speed vector Figure 1. This angle is normally calculated from the GNSS data, and it is crucial to establish the Actual Tracking Angle (ATA). There is also a crab-angle that is a resultant of the wind drift, and these two values are strictly correlated with each other [17]. Figure 2 reveals the difference between drift angle and crab angle Figure 2.

If a GNSS data loss occurs, only the drift must be estimated with use of another source. One of the solutions could be vision-based motion estimation, since it provides more versatile in integrating it into the UAV navigation system. It seems that the optical flow algorithms are the most popular approach for estimating the motion of a vehicle. There are some research that use optical flow and embedded camera to estimate the speed of a moving robot [18,19].

Taking into account that in the considered the scenario a crucial parameter to estimate is the MUAV drift, the most appropriate approach will be motion estimation using optical flow or a similar computer vision algorithm. The main goal was to create a system that can estimate the drift data with a minimal calculat ing effort for the processor. That excluded use of the deep learning object recognition or other highly advanced computer vision algorithms. Our second goal was not to use any additional sensors besides an embedded camera. Embedded cameras or opto-electronic heads are pretty common on board of MAVs. Using something that common as camera is useful for upgrading the existing MUAV products already that are delivered to the customers only by updating their software or changing the camera lens. It is especially significant in the case when any necessary hardware upgrades would be economically unjustified or even impossible to be carried out. This kind of approach is consistent with MUAV characteristic as a really cheap multipurpose aerial vehicle. The idea was to use an embedded fish-eye camera that was located on the fuselage belly. By using the digital stabilization and image cropping, we were able to observe normal to the flat ground area directly under the MUAV in real time, regardless of the maneuvers performed by the plane. Theembedded camera takes a snapshot of the reference area while simultaneous ly observing the small part of it in real time. By using Template Matching (TM) algorithms for finding the best match of the template on our base image, we were able to calculate the wind drift of the vehicle.

In [20], the authors present a similar method to the one followed in this paper. The major functions of that research was to track the landing spot with no marker and provide the position and velocity data to replace the existing GNSS measurement in a UAV control system. To achieve that, they used the optical flow to estimate the landing speed and position in relation to the reference image frame. They also used an embedded facing down camera. However, they used a quadcopter instead of fixed-wing plane also, the speed estimation method was different. The problem of fixed-wing motion estimation was described in [21]. In that work, the authors use the facing down optical flow sensor mounted on an MUAV fuselage belly to estimate the crab-angle. The wind velocity was computed by combining the crab-angle with measurements of ground track from GPS and the MUAV’s airspeed.

The presented research, in contrast to the aforementioned works, uses Template Matching vision based algorithms instead of optical flow. TM was used to simplify the algorithms and to decrease the propable processing cost. Other differences were: using an embedded camera instead of an optical flow sensor, windspeed calculation methods, an image stabilization method and overall purpose for the drift estimation. The remainder of this paper is organized, as follows. Section 2 presents the general GSOE computation process. Section 3 shows the simulation design, implementation, and results of using the SIL system of the Simulink dynamic model of UAV with FlightGear visualization. Section 4 shows the hardware design and flight test results using a fixed-wing MUAV with an embedded fish-eye camera. Finally, Section 5 gives the conclusions of this study.

## 2. GSOE Algorithms and Methods

**Figure 3:**
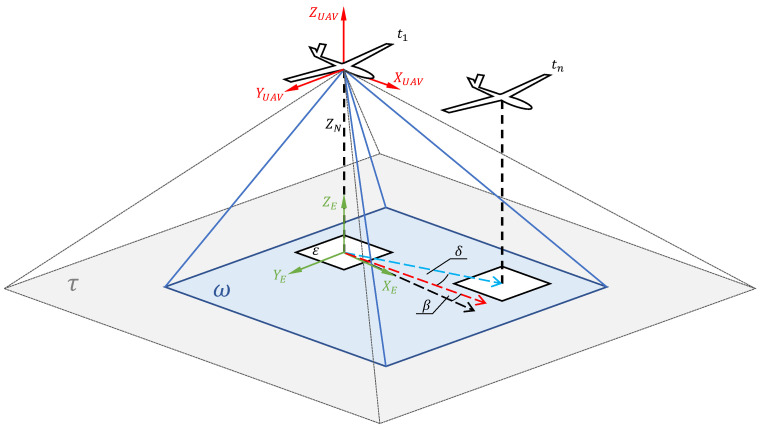
Coordinate system with template matching scheme.

**Figure 4:**
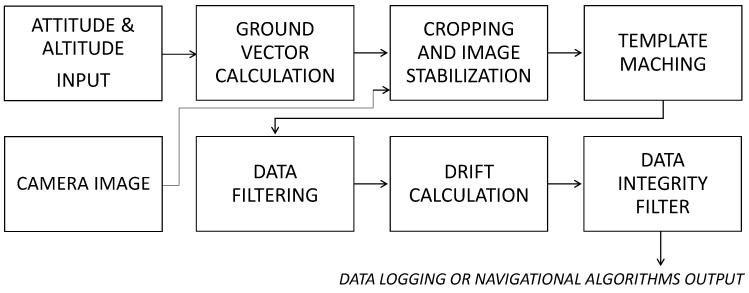
Flow chart of the GSOE.

The concept of GSOE design is to compute the translocation of the aerial vehicle relative to the ground. This is obtained by the observation of area normal to the flat earth under the MUAV. Figure 3 shows the graphical representation of the concept. An aircraft coordinate system $X_{UAV}$; $Y_{UAV}$; $Z_{UAV}$ has six degrees of freedom. The flat earth coordinate system is represented by $X_{E}$; $Y_{E}$;$Z_{E}$ and it is fixed in the starting point of the mission. Plane τ represents the fish-eye embedded camera field of view. Plane τ is fixed with aircraft coordinate system. FoV must be greater than the maximum pitch and roll range. Due to this, we can observe the ground area directly under the aircraft, regardless of its attitude. Because of fish-eye lens distortion (barrel distortion), we must identify the camera’s implicit parameters and calibrate the image. Using the real time attitude and altitude data, we can calculate the normal vector $Z_{N}$ from the camera focal point to the ground. After that, we can crop the image that is represented by ω, that lies on the $X_{E}$; $Y_{E}$ axes. We will use ω a snapshot image as a base for TM algorithm. The template is generated by cropping the smaller area that is represented by ε. Template sampl ing rate is crucial in achieving robustness and effectiveness of the algorithm. A lower sampling rate decreases the calculating effort, but increases the error at the lower altitude Above Ground Level (AGL). With a higher sampling rate, we can decrease the error, but increase the calculation effort which may result in potential delays and desynchronizations. The TM process is carried out cyclically during the template (ε) migration in time $t_{1}-t_{n}$. A new cycle starts when the template reaches a defined critical position, time, or filter values. By observing the template shifting on the base image, we can calculate the ground speed components, the drift of the MUAV or even the wind direction and speed. A data integrity filter was created to evaluate the estimated parameters before logging them or releasing to the autopilot. Figure 4 was created for better understanding of the GSOE design and algorithm flow.

### 2.1. Camera Calibration and Distortion Correction

Classical calibration methods were used in order to obtain the camera matrix. Calibration is performed once on the airplane assembling stage when a camera is already mounted inside the fuselage. The method is based on a chess board pattern of a known size to estimate the camera intrinsic parameters and the lens distortion coefficients [22]. Based on the pinhole camera model, the projection matrix is:

(1) $$C=\left|\begin{array}{ccc} f_{x} & 0 & p_{x}\\ 0 & f_{y} & p_{y}\\ 0 & 0 & 1 \end{array}\right|$$

where $f_{x}$ and $f_{y}$ are the camera focal lengths, and $p_{x}$ and $p_{y}$ represent the optical center in pixel coordinates. The wide FoV, which is necessary to counteract aircraft attitude changes, introduces barrel distortions into image. The first step of image preparation is a distortion correction. Because this is a standard process [23], I do not review this method.

### 2.2. Ground Vector Calculation

In order to calculate the normal ground vector, we need information about our attitude, altitude, and some camera parameters such as an image size, a horizontal field of view (HFOV), and a vertical field of view (VFOV). For camera stabilization and cropping what we really need is location of the point where normal ground vector intersect with image plane:

(2) $$X_{stab}=\frac{X_{px}}{2}*\left(\frac{tan\phi }{tan(\frac{HFOV}{2})}+1\right)$$

(3) $$Y_{stab}=\frac{Y_{px}}{2}*\left(\frac{tan\theta }{tan(\frac{VFOV}{2})}+1\right)$$

where $X_{stab}$ and $Y_{stab}$ are intersect point with image plane, $X_{px}$ and $Y_{px}$ are image size in pixels, φ is roll angle, θ is pitch angle.

### 2.3. Cropping and Stabilization of Image

The intersection point is a center of the base image that we must crop from the calibrated image. Cropping of that source image works as stabilization due to the maneuvers of the aircraft. In this case, it was assumed that a raw camera images have the 800 × 600 px size, and the roll angle ranges from $-45^{\circ }$ to $45^{\circ }$, the pitch angle ranges from $-20^{\circ }$ to $20^{\circ }$, HFOV is $110^{\circ }$ and VFOV is $87.5^{\circ }$. The se parameters and the template matching calculation effort have an impact on the base image size. The simulation tests determined the base image optimal size as 120 × 72 px and the template size as 18 × 18 px. In the performed simulations, the template cropping sample rate varies from 25 Hz to 60 Hz. As an optimal sample rate, 30 Hz was chosen for simulation purpose and 25 Hz for flight tests.

### 2.4. Template Maching

In order to perform template matching on the cropped images, it is necessary to perform color space conversion from the camera original $R^{\prime }G^{\prime }B^{\prime }$ to intensity [24]:

(4) $$intensity=\left[0.299\;0.587\;0.114\right]\left[\begin{array}{c} R^{\prime }\\ G^{\prime }\\ B^{\prime } \end{array}\right]$$

The TM algorithm finds the best match of the template within an input image. The algorithm computes match metric values by shifting the template over a region of interest or the entire image, and then finds the best match location. Because this algorithm is well known and described in many scientific works [25], I do not review this method. The algorithm outputs either the match metric values or the one-based (*x*,*y*) coordinates of the best template match. The sum of squared differences (SSD) algorithm was chosen. SSD is one of the measure match that based on pixel-by-pixel intensity differences between template and base image [26]. SSD calculates the summation of squared for the product of pixels subtraction between two images [27]. The matching point is determined by considering the minimum value in the image matrices. SSD is directly while using the formulation of sum of square error. In the digital form the SSD equation is:

(5) $$SSD\left(i,j\right)=\sum_{i=1}^{R}\sum_{j=1}^{C}{\left(f\left(i,j\right)-g\left(i+u,j+v\right)\right)}^{2}$$

where *R* is size the of rows in the base image and *C* is the size of columns, while *u* and *v* are variables, shift components along *x*-direction and *y*-direction, respectively [28].

The applied TM algorithm [29] implements two different searching methods: Exhaustive or Three-step. The Exhaustive (or full search) search method is computationally intensive, because it searches at every pixel location of the image. However, this method provides a more precise result. The Three-step search method is a fast search that uses a neighborhood approach versus a search at every pixel. Naturally, there are a variety of different motion estimation algorithms, such as the Four-step search or the Diamond search algorithm. The choice of the method depends on the computing power available. The Exhaustive was chosen because applied algorithm only has two methods available and the computing power of the on-board computer was sufficient.

### 2.5. Data Filtering

As it was mentioned in Section 2.4, the image template is matched to the base image n-times in cycle. The filter analyzes the incoming data pattern from each cycle to ensure that the last data point in the cycle is valid and consistent with the previous steps. If the filter detects an anomaly, it ends the cycle and a new base image will be selected. The data filtering algorithm could be expressed as follows:

(6) $$|L_{X}\left(n\right)-L_{X}\left(n-1\right)|\geq \xi \;\wedge \;|L_{Y}\left(n\right)-L_{Y}\left(n-1\right)|\geq \xi$$

where $L_{X,Y}$ are template location on the base image, *n* is matching step, n−1 is one step delay, and ξ is filtering threshold.

### 2.6. Drift Calculation

The TM output is the location of the template center at the base image, where the left top corner is the origin of the coordinate system. Directly from that output we can calculate the displacement of the MUAV relative to the base image:

(7) $$\Delta X=X_{0}-L_{X}$$

(8) $$\Delta Y=L_{Y}-Y_{0}$$

where $X_{0}$ and $Y_{0}$ are the locations of the TM starting point on the base image. This point represents the focal point of the lens. Now, we can calculate the drift of the MUAV:

(9) $$\delta =atan(\frac{\Delta X}{\Delta Y})$$

Unmistakably, with an estimated drift, we can calculate a lot of different flight parameters. First, we must establish the actual pixel size in SI values:

(10) $$x_{m}=h*tan\left(0.5*HFOV\right)*\frac{x_{px}}{X_{px}}$$

(11) $$y_{m}=h*tan\left(0.5*VFOV\right)*\frac{y_{px}}{Y_{px}}$$

where $x_{px}$ and $y_{px}$ are the template matrix size and *h* is the altitude. Next, using IMU and the Autopilot data, we can calculate the Actual Tracking Angle (ATA):

(12) ATA=β+ψ+δ

where β is the sideslip angle and ψ is the true heading. We can calculate the wind speed and its direction:

(13) $$W_{s}+W_{d}i=A+Bi-C*Di$$

where *A*, *B*, *C*, *D* are:

(14) A=|TAS|∗cos(β+ψ)

(15) B=|TAS|∗sin(β+ψ)

(16) C=|GS|∗cos(ATA)

(17) D=|GS|∗sin(ATA)

where $W_{s}$ is the wind speed in $\left[\frac{m}{s}\right]$, $W_{d}$ is the wind direction in [deg] clockwise from North, GS is ground speed in $\left[\frac{m}{s}\right]$, and TAS is the true airspeed.

### 2.7. Data Integrity Filter

Since TM reliability depends on the input image quality and the image content, it sometimes happens that the algorithm can output invalid data. This can happen, on a consistent terrain, such as grass fields, desert, overcalm water, etc. First, the filter should detect that, but it then starts a new cycle and passes the last value before restarting. That is why there is a data integrity filter comparing the data from the last cycles and it will erase the inconsistent data. Of course, the efficiency of this filter is correlated with the number of past cycles to compare and the maximum delay that we can afford. The operating method is similar to the mentioned data filter shown in Section 2.5.

### 2.8. GNSS Drift Calculation

As the simulation and flight test results were compared to the GNSS data, it is worth mentioning how the GNSS drift is calculated by the autopilot [30]. These GNSS drift calculation algorithms are not part of the GSOE, but they are usually a part of the autopilot itself. In order to compare the results a new Simulink model was created. It uses the logged data to calculate the GNSS drift, plot the top view of the flight path, and to plot the drift in the heading function. First, the Simulink Aerospace toolbox LLA to Flat Earth block [31] was used to estimate the flat Earth position from the geodetic latitude, longitude, and altitude. Second, with the MUAV position in the Cartesian coordinates, we can calculate the position displacement:

(18) $$\Delta X_{GNSS}=X_{GNSS}\left(n\right)-X_{GNSS}\left(n-1\right)$$

(19) $$\Delta Y_{GNSS}=Y_{GNSS}\left(n\right)-Y_{GNSS}\left(n-1\right)$$

where $X_{GNSS}$ and $Y_{GNSS}$ denote the positions in Cartesian coordinates. Next, we can take into account the MUAV heading to calculate the drift components:

(20) $$\left|\begin{array}{c} \delta_{x}\\ \delta_{y} \end{array}\right|=\left|\begin{array}{cc} cos\psi & sin\psi \\ -sin\psi & cos\psi \end{array}\right|\left|\begin{array}{c} \Delta X_{GNSS}\\ \Delta Y_{GNSS} \end{array}\right|$$

Now, we can calculate the *GNSS* based drift:

(21) $$\delta_{GNSS}=atan\left(\frac{\delta_{y}}{\delta_{x}}\right)$$

## 3. Software in the Loop

The simulations of GSOE were conducted in the SIL in order to examine the feasibility and performance of the developed algorithms. The simulation was developed in Simulink and it contains the MUAV Plant model, Environment, Autopilot, Logic, and navigational algorithms. The FlightGear software was used for visualization purpose, and for creating a camera model. The image from the FlightGear was sent back to the same Simulink model, where the GSOE was also implemented.

### 3.1. Simulation Design

**Figure 5:**
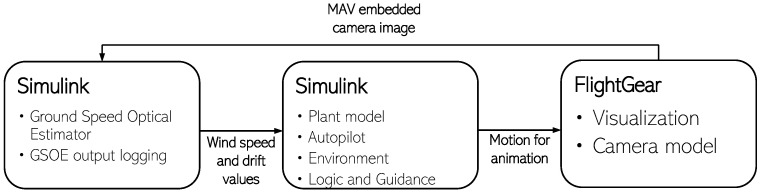
SIL loop.

The Simulink communicates with the FlightGear via the User Data Protocol (UDP). The FlightGear simulator was used for the visualization of the flight and for simulating the MUAV embedded camera image. An embedded belly camera model was created. The camera used had $110^{\circ }$ HFOV and was located facing down on the MUAV fuselage belly in the center of gravity (CoG). Figure 5 shows the simulation loop design.

The prepared Simulink model contains several blocks to best simulate the environment and flight dynamics. First, the MUAV plant based on the 6DoF (Quaternion) aircraft dynamic calculation was implemented. Subsequently, autopilot was created, containing LOGIC system blocks, GUIDANCE blocks, and STABILIZATION blocks. Lastly, environment was created, which includes the WGS84 gravity model, COESA atmosphere model, and complex wind model composed of a wind shear model, Dryden wind turbulence model, and discrete wind gust model.

A flight with multiple turns, wind speed and directions, as well as altitude and flight speed changes was simulated. The GSOE algorithms were tested on varying FlightGear Earth textures (maps). The real time drift estimation was logged. The MATLAB script was created to extrude the logged drift in the heading function. Subsequently, heading was rounded to whole number and, for heading from $1^{\circ }$ to $360^{\circ }$, median drift values were calculated. Due to that, we were able to evaluate a GSOE performance independently of the flight duration and trajectory.

### 3.2. Simulation Results

**Figure 6:**
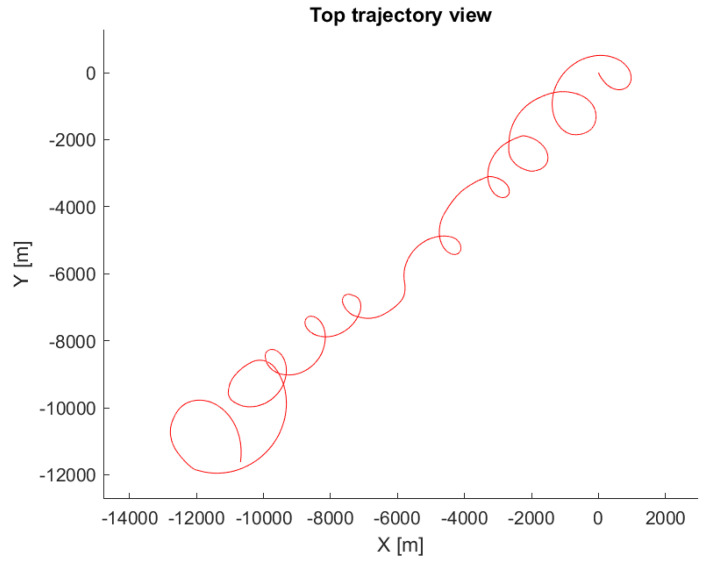
Flight trajectory top view.

**Figure 7:**
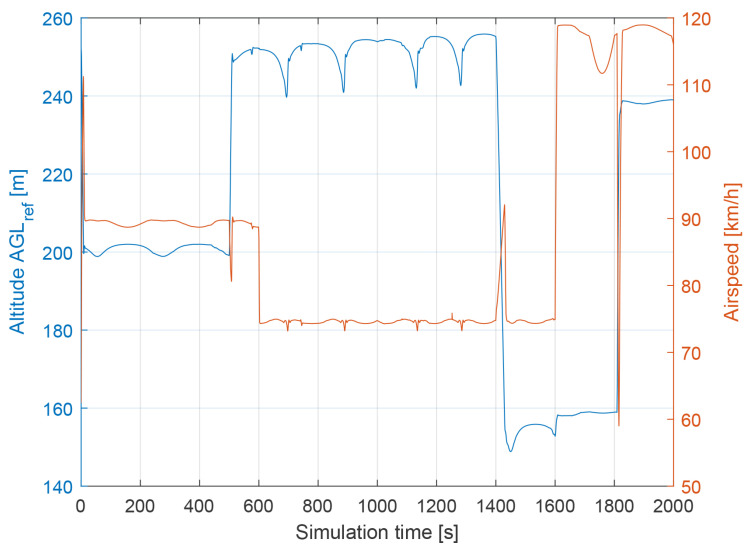
The altitude above ground level with respect to the reference point and airspeed as a function of simulation time.

**Figure 8:**
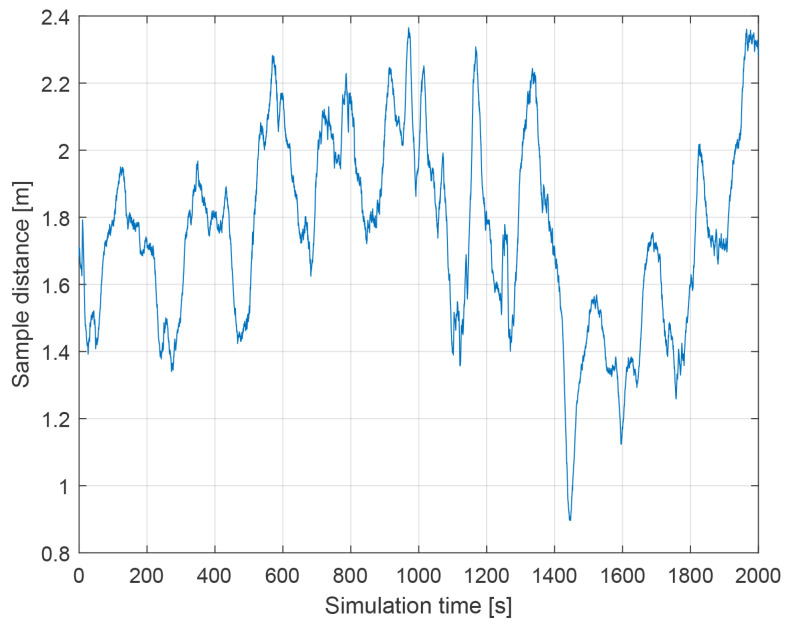
Sample distance as a function of simulation time.

The following conditions were set during the presented simulation:

- Wind speed at 6 m altitude: 5 $\frac{m}{s}$
- Wind direction at 6 m altitude (clockwise from north): $45^{\circ }$
- Flight trajectory: presented at Figure 6
- TAS: presented in Figure 7
- Altitude above ground level in respect to the reference point: presented at Figure 7. Ground reference point is the last known ground elevation before MUAV lost *GNSS* data.
- Simulation time: 2000 s
- Camera HFOV: $110^{\circ }$
- Camera matrix size: 800 × 600 px
- Base image size: 121 × 73 px
- Template image size: 19 × 19 px
- GSOE sample time: 30 Hz
- GSOE sample distance: presented at Figure 8

**Figure 9:**
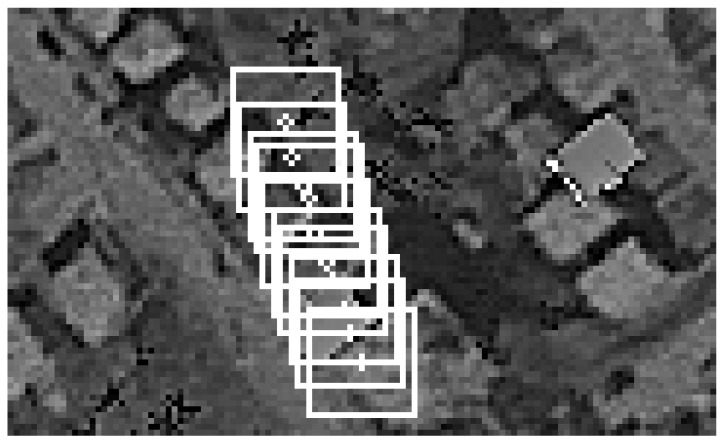
Simulation results. Template matching (TM) cycle merged into one image.

Apart from the drift, base images and template images from each cycle wew also logged. One of the TM cycles was merged into one picture and presented in Figure 9.

**Figure 10:**
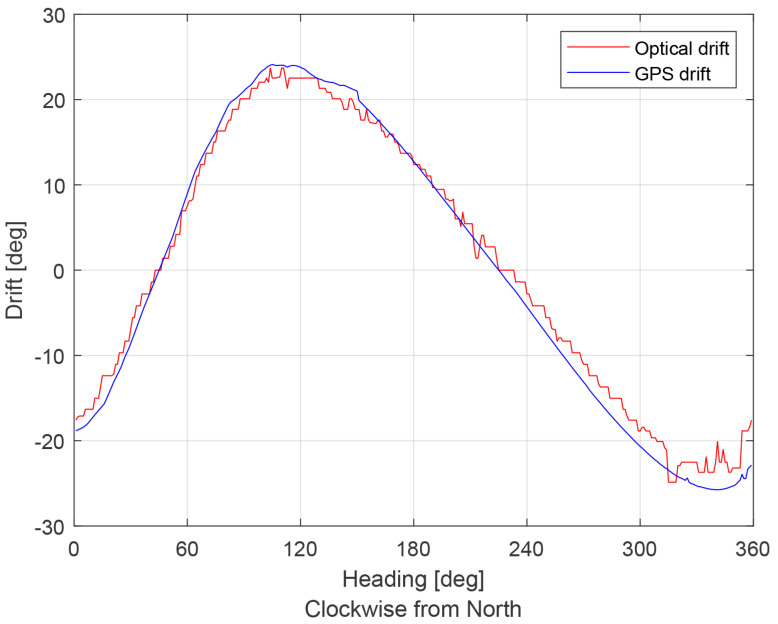
Simulation results. Comparison Global Navigation Satellite System (GNSS) drift and optical estimated drift.

Figure 10 presents the simulation results. The GSOE optical drift estimation was compared to the simulated GNSS drift calculation (the MUAV position in the Cartesian coordinate system with noise added). A comparison of the simulation results shows that the proposed GSOE estimate drift angle is very close to the GNSS drift values. The mean absolute error between drift values is $0.9603^{\circ }$.

## 4. Flight Test

Because accurate drift measurements mostly depend on a camera image and TM algorithms efficiency, the GSOE was implemented as a stand-alone application that was running during the flight.

### 4.1. Flight Test Hardware

**Figure 11:**
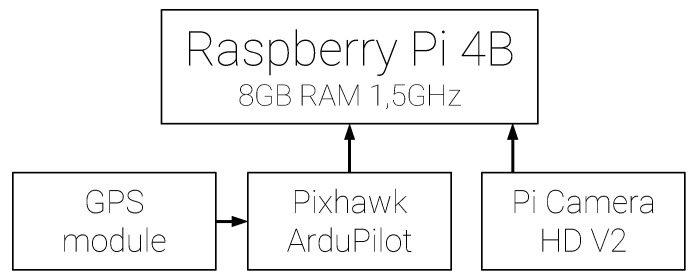
GSOE hardware scheme.

**Figure 12:**
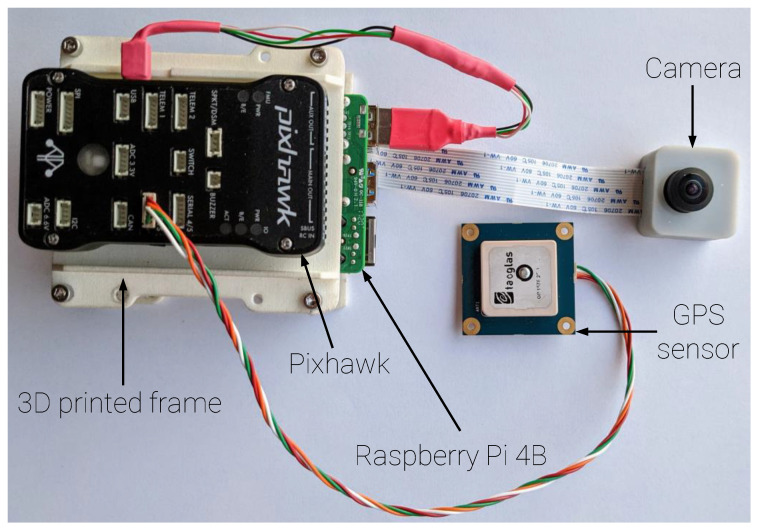
GSOE flight test hardware.

Because of its availability, popularity, and compatibility with the Simulink, the Raspberry Pi 4B 8 G RAM 1.5 GHz was selected as an on board computer. The GSOE Simulink model was uploaded to computer as a stand-alone application via the Simulink^®^ Support Package for Raspberry Pi™. For extra security, AHRS data were not taken from MUAV Autopilot, but a second independent Pixhawk unit was used. It had modified ArduPilot firmware. Firmware was modified in order to specify that only necessary data will be stream ed via the MUAVlink Router. Additionally, the stream rate was increased to 300 Hz for IMU data. Such high value should provide smooth camera stabilization and cropping. The GNSS u-blox module with the Taoglas antenna was connected to the autopilot and then Pixhawk to on-board computer. A Pi Camera HD v2 was connected directly to the on-board computer. To provide fish-eye capability, the original lens was changed to SONY IMX219 8 MP. The Raspberry and Pixhawk were mounted onto the SLS 3D printed frame. Additionally, the camera had a SLA 3D printed case, designed specifically for this test. Figure 11 and Figure 12 present the hardware scheme and picture.

**Figure 13:**
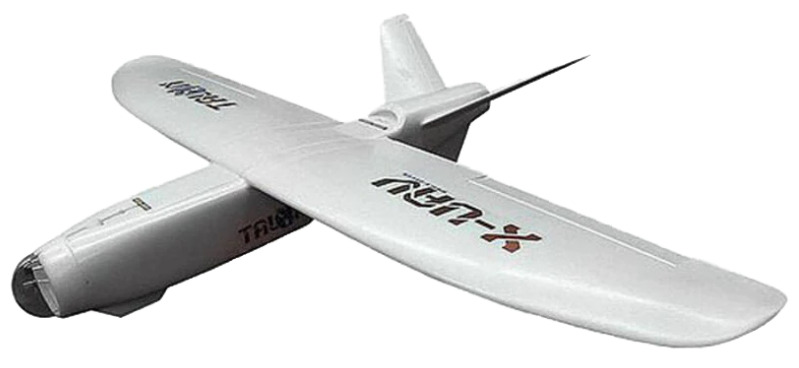
X-UAV Talon. Plane used for test in flight.

As an MUAV platform the X-UAV Talon Figure 13 was chosen. Talon is a fixed-wing UAV in a conventional layout with a high wing configuration and a V-tail. The frame was mounted inside the fuselage payload bay, near the CoG point. The camera was installed on the fuselage belly directly in the CoG. To achieve that, an FDM 3D printed camera mount was designed and built into the plane structure.

A flight with multiple turns, altitude, and flight speed changes was made. The real time drift estimation, camera image, GNSS, and AHRS data were logged.

### 4.2. Flight Test Results

**Figure 14:**
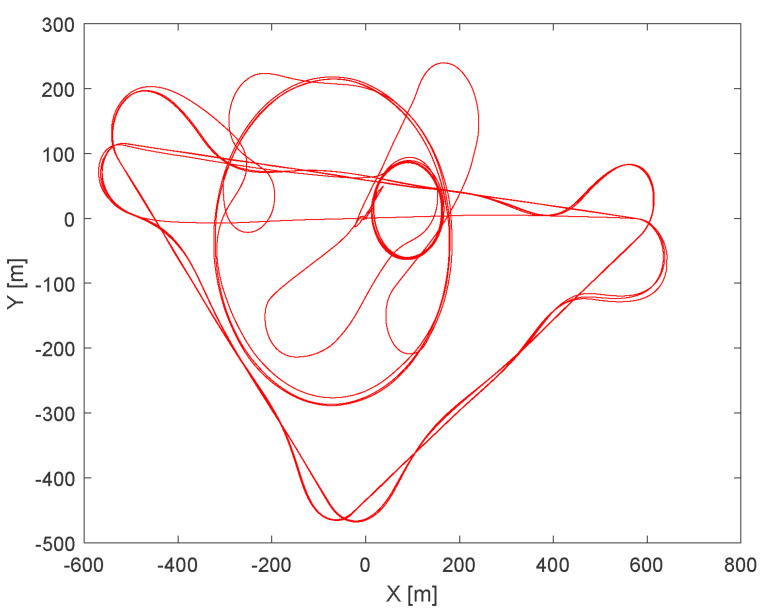
Flight trajectory top view.

**Figure 15:**
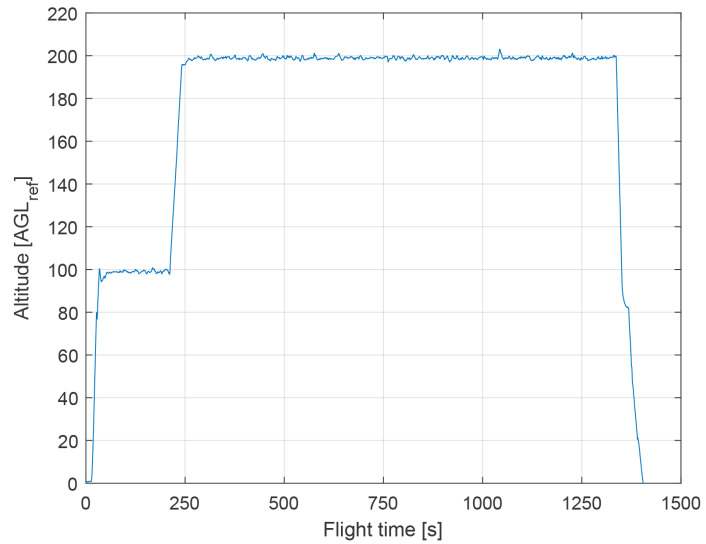
Altitude above ground level in respect to the reference point as a function of simulation time.

The following conditions were present during the flight test:

- Wind speed at 6 m altitude: 1.5$\frac{m}{s}$
- Wind direction at 6 m altitude (clockwise from north): $180^{\circ }$
- TAS: 75 $\frac{\mathrm{km}}{h}$ (maintained during cruising)
- Flight trajectory: presented in Figure 14
- Altitude above ground level in respect to the reference point: presented at Figure 15. Ground reference point is the last known ground elevation before MUAV lost GNSS data.
- Mission time: 1440 s
- Camera HFOV: $110^{\circ }$
- Camera matrix size: 800 × 600 px
- Base image size: 121 × 73 px
- Template image size: 19 × 19 px
- GSOE sample time: 25 Hz

**Figure 16:**
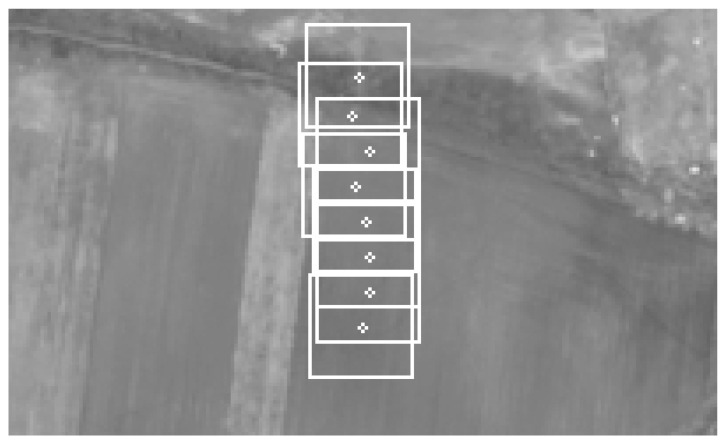
Flight test results. TM cycle merged into one image.

One of the TM cycles was merged into one picture and presented in Figure 16.

**Figure 17:**
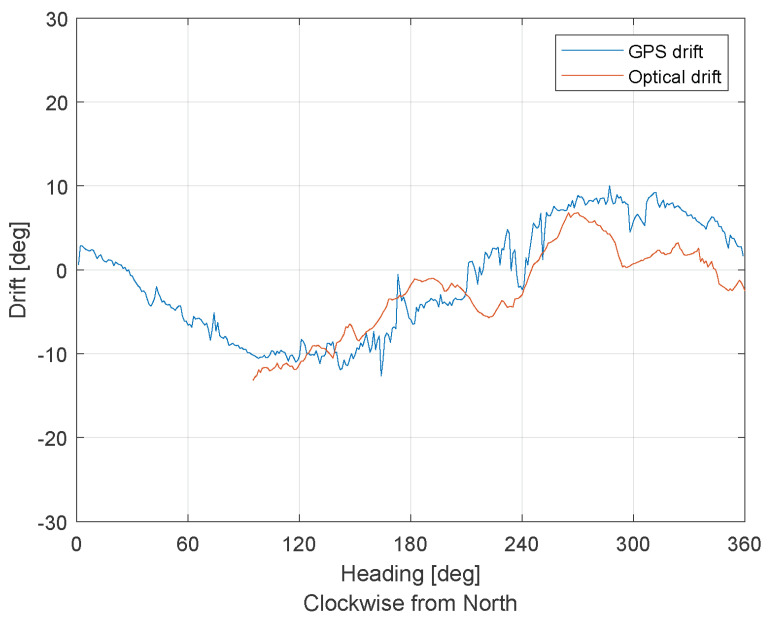
Flight test results.

Figure 17 presents the flight test results. The GSOE optical drift estimation was compared to the GNSS drift calculation. A comparison of the flight results shows that the GSOE was not that effective as it was in the SIL test. The mean absolute error between the drift values is $3.5103^{\circ }$. The GSOE filtered drift data from $0^{\circ }$ to $94^{\circ }$ of heading values. The data were deleted by the integrity filter, probably because of poor TM estimation in that heading range.

## 5. Conclusions

This paper introduces a system for drift estimation with use of GSOE algorithms. The simulation results showed that the implementation of the proposed GSOE in various flights maneuvers, such a turns (+/−$45^{\circ }$ of roll), altitude changes (with +/−$20^{\circ }$ of pitch), airspeed changes, and flight conditions was successfully validated in the SIL simulation. The mean absolute error between the GNSS estimated drift and the optical drift never was bigger than $1^{\circ }$. From a comparison of the SIL results to the flight test results, we could notice the performance and efficiency drop. This was probably related to the camera blur during the flight maneuvers. There were some delays that were difficult to remove because stabilization and cropping algorithms depend on Pixhawk IMU data. In fast turns or turbulent atmosphere, the camera image became blurry for a split second. To solve this problem, future work may involve replacing the Raspberry Pi Camera with a built-in IMU Camera.

The GSOE can also be used to estimate the wind speed and direction or to navigate without the GNSS. This is a promising topic of future research, since MUAV’s may have to navigate in the case of the GNSS failure, jamming, or unavailability.

## Data Availability

Not applicable.

## Abbreviations

The following abbreviations are used in this manuscript:

GSOE	Ground Speed Optical Estimator
UAV	Unmanned aerial vehicle
MUAV	Miniature unmanned aerial vehicle
IMU	Inertial measurement unit
AHRS	Attitude and heading reference system
GNSS	Global Navigation Satellite Systems
LIDAR	Light detection and ranging
AI	Artificial Intelligence
SLAM	Simultaneous localization and mapping
TM	Template matching
ATA	Actual tracking angle
FOV	Field of view
HFOV	Horizontal field of view
VFOV	Vertical field of view
SSD	Sum of squared differences
GS	Ground speed
TAS	True airspeed
LLA	Geodetic latitude, longitude, and altitude coordinate system
SIL	Software in the loop
UDP	User data protocol
CoG	Center of gravity
WGS84	World Geodetic System ’84
COESA	Committee on Extension to the Standard Atmosphere
AGL	Altitude above ground level
$AGL_{ref}$	Altitude above ground level in respect to the reference point
SLS	Selective laser sintering
SLA	Stereolithography apparatus
FDM	Fused filament fabrication
